# The association of low penetrance genetic risk modifiers with colorectal cancer in lynch syndrome patients: a systematic review and meta-analysis

**DOI:** 10.1007/s10689-017-9995-8

**Published:** 2017-05-15

**Authors:** Neil Donald, Salim Malik, Joshua L. McGuire, Kevin J. Monahan

**Affiliations:** 1 Faculty of Medicine, Imperial College London, London, UK; 2 Family History of Bowel Cancer Clinic, West Middlesex University Hospital, Chelsea and Westminster Hospitals NHS Trust, London, UK

**Keywords:** Colorectal cancer, Lynch syndrome, HNPCC, Polymorphisms, Low penetrance, Genetic risk, Systematic review, Meta-analysis

## Abstract

Lynch syndrome (LS) is a highly penetrant inherited cancer predisposition syndrome accounting for approximately 1000 cases of colorectal cancer (CRC) in the UK annually. LS is characterised by autosomal dominant inheritance and germline mutations in DNA mismatch repair genes. The penetrance is highly variable and the reasons for this have not been fully elucidated. This study investigates whether low penetrance genetic risk factors may result in phenotype modification in LS patients. To conduct a systematic literature review and meta-analysis to assess the association between low penetrance genetic risk modifiers and CRC in LS patients. A systematic review was conducted of the PubMed and HuGENet databases. Eligibility of studies was determined by pre-defined criteria. Included studies were analysed via the per-allele model and assessed by pooled odds ratios and establishing 95% confidence intervals. Study heterogeneity was assessed via Cochrane’s Q statistic and I2 values. Publication bias was evaluated with funnel plots. Subgroup analysis was conducted on gender. Statistical software used was the Metafor package for the R programme version 3.1.3. Sixty-four polymorphisms were identified and sufficient data was available for analysis of ten polymorphisms, with between 279 and 1768 CRC cases per polymorphism. None demonstrated association with CRC risk in LS patients. However in sub-group analysis the polymorphism rs16892766 (8q23.3) was significant in males (OR 1.53, 95% CI 1.12–2.10). The variable phenotype presentation of the disease still remains largely unexplained, and further investigation is warranted. Other factors may also be influencing the high variability of the disease, such as environmental factors, copy number variants and epigenetic alterations. Investigation into these areas is needed as well as larger and more definitive studies of the polymorphisms analysed in this study.

## Introduction

Colorectal cancer (CRC) development is highly complex and multi factorial involving lifestyle, environmental and genetic factors. The concordance rates of cancer in monozygous and dizygous twins suggest that about 35% of the variation in cancer risk might typically be ascribed to heritable factors [1]. Highly penetrant (Mendelian) genetic syndromes account for 5–10%, the most common of which is Lynch syndrome (LS), previously known as Hereditary Non-Polyposis Colorectal Cancer (HNPCC) [2–4]. The lifetime risk of development of CRC in LS is 28–75% in men and 24–52% in women [5].

LS is inherited in an autosomal dominant pattern and is due to either germline or epigenetic (such as via the EPCAM gene) mutations in one or more of the DNA mismatch repair genes (MMR) [6–8]. MMR genes act to correct base–base mismatches and insertion/deletion loops that occur during DNA replication and recombination [9]. Mutations subsequently result in S-phase replication errors that cause micro-satellite instability and have the potential to drive tumourgenesis [10]. Four main MMR genes have been implicated in the development of LS—*MLH1* and *MSH2* which together account for 70–80% of cases, *MSH6* which is approximately 20% and *PMS2* which is associated with lower penetrance [11].

These mutations cause accelerated carcinogenesis through the adenoma-carcinoma sequence [7]. In addition, in patients with LS, CRC development has a younger age of onset (average age of 45 years) with 10% of CRC cases before 50 years [6, 12].

It has been reported that unrelated families with the same mutation often present with widely variable disease profiles, and even within families that have the same mutation, age of onset, disease progression and prognosis can be unpredictable. The reason for this has not been established; it has been hypothesised that this difference in disease expression may be due to individuals harbouring multiple low penetrance genetic modifiers, environmental exposures, or a combination of both [13]. Bodmer and Bonilla have proposed that this variation may be due to low penetrance allelic variants that are commonly found in the population which only very slightly increase risk individually, however work synergistically to increase overall risk [14].

With the introduction of genome-wide association studies (GWAS) it has become possible to evaluate the role of common low penetrance genetic modifiers and how they can affect the variable disease expression that occurs both within and between families or individuals with similar MMR gene profiles.

Previous studies have shown the impact of low-penetrance genetic modifiers in CRC, however there has been no meta-analysis of these in relation to LS patients [15]. Alternatively the genotype-phenotype correlation may be primarily due to the penetrance related to the primary germline mutation in MMR genes. Therefore our aim was to conduct the first systematic literature review and meta-analysis to evaluate the role and effects of common low penetrance genetic polymorphisms in modifier genes with regard to a better understanding of their association with CRC development risk in patients with LS. This would in our opinion lead to more effective patient counselling with specific genetic profiles on their disease prognosis and course.

## Methods

The literature search and meta-analysis were conducted in line with the Preferred Reporting Items For Systematic Review and Meta-Analysis Protocols (PRISMA-P) 2015 statement [16].

### Study search

A literature search of the PubMed database was undertaken from January 2000 through August 2016. A first search was conducted using the terms “Genetic polymorphisms” AND “Lynch syndrome” OR “HNPCC” OR “Hereditary Nonpolyposis Colorectal Cancer”.

Once a specific gene or polymorphism was identified a subsequent search was done using the terms “rs number” OR “[gene name] polymorphism” AND “Lynch syndrome” OR “HNPCC” OR “Hereditary Nonpolyposis Colorectal Cancer”.

An additional search of the HuGENet database was carried out (last search August 2016) using the terms “Gene name” AND “Lynch syndrome”.

Where a gene has two commonly used names both were used during the literature search. The results obtained from the second search were integrated with the first search results.

Further relevant articles were studied from the bibliographies of eligible articles and previously conducted meta-analysis that looked at polymorphisms related to CRC to identify any further relevant studies to be included.

**Fig. 1:**
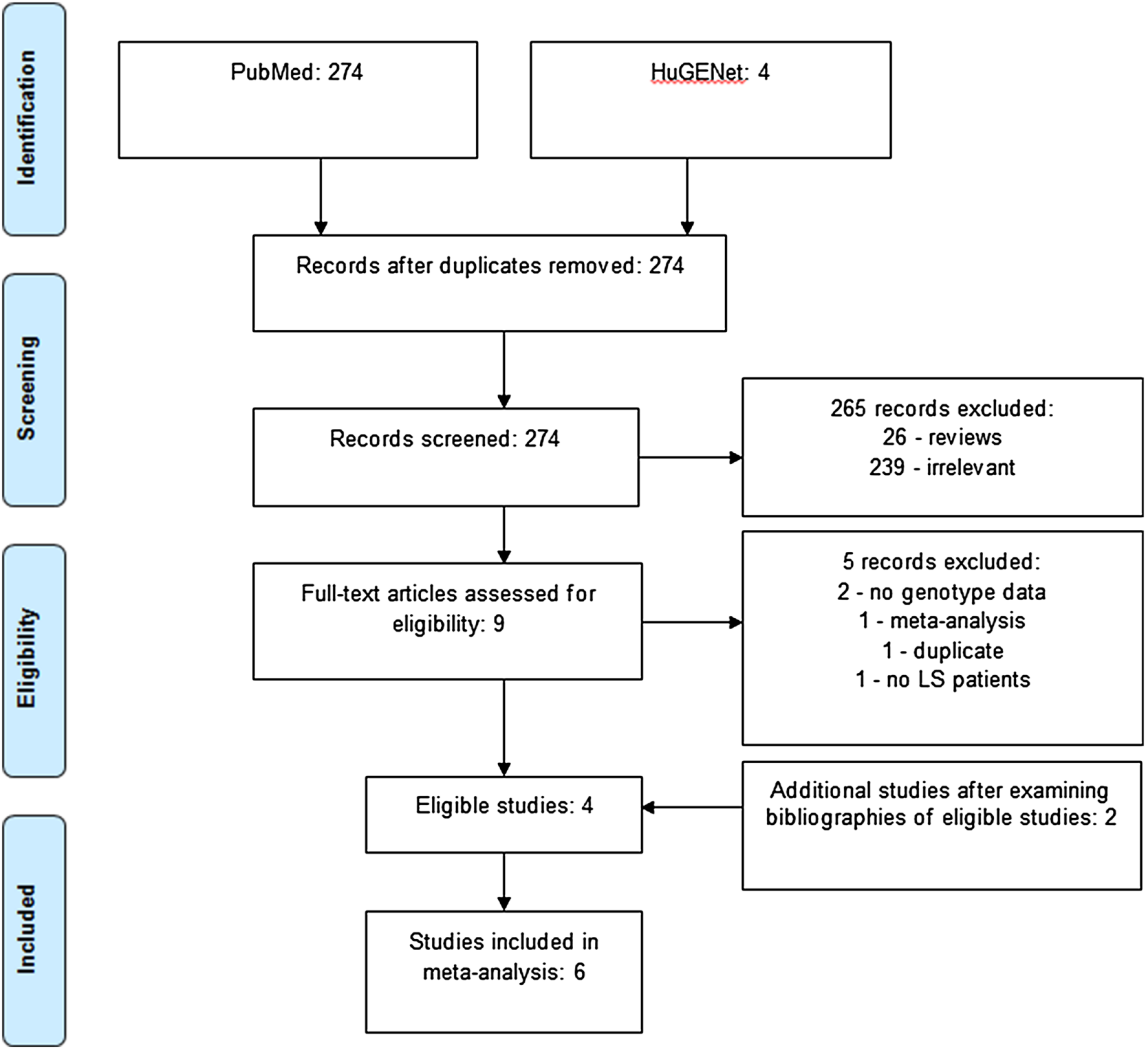
Flowchart showing selection process for the polymorphism rs9344 in the gene *CCND1*

An example by which studies were included through screening and determining eligibility is shown in Fig. 1.

Whether or not the study was deemed eligible for inclusion was based upon pre-defined inclusion and exclusion criteria and by mutual agreement. Studies had to meet ALL the inclusion criteria and were excluded if they met ANY of the exclusion criteria as show in Table 1.

**Table 1:** Table showing predefined inclusion and exclusion criteria

Inclusion	Exclusion
Examines the relationship between a low penetrance genetic risk modifier and colorectal cancer in Lynch syndrome	Case or review studies
Case control study	Studies with insufficient data to determine OR
At least 40 cases and 40 controls	Animal or tissue based studies
Study participants have been randomly selected	Duplicate studies (use of same study participants and data but published in two different papers)
Confirmed diagnosis of Lynch syndrome either through clinical criteria or mutations to MMR genes	Control population is not in Hardy–Weinburg equilibrium
Confirmed diagnosis of colorectal cancer	
Full article published in English in a peer reviewed journal	

### Data extraction

For included studies, data extraction and quality appraisal was performed by one reviewer (ND) and checked by a second (KM). Any disagreements were resolved by discussion, with involvement of a third reviewer as necessary.

Once a study was deemed eligible for inclusion the following data was acquired using a standardised database: Name of lead author, publication year, paper title, polymorphism or polymorphisms investigated, minor allele frequency, number of cases, number of controls, genotype frequencies for cases, genotype frequencies for controls, genotype frequencies for MMR mutation subtypes for cases and controls, genotype frequencies for male and female cases and controls, diagnosis method of cases and controls, country of study origin and ethnicity of study participants.

### Statistics

Statistical analysis was undertaken using the Metafor package in R (version 3.1.3) [17].

Hardy–Weinberg equilibrium (HWE) was assessed using the Pearson’s Chi-squared test which tested for variation between the expected genotype frequencies and the observed frequencies. A value above 3.84 (p < 0.05) was regarded as being statistically significant for variation between the two frequencies. All included studies were calculated to be in HWE.

Quantitative synthesis was achieved by investigating pooled odds ratios (OR), and 95% confidence intervals (CI). The allele specific model was utilized in OR calculations. Polymorphisms were found to be statistically significant if the 95% CI did not cross 1.

Study heterogeneity was measured with Cochrane’s Q statistic and I² values. Guidelines suggest that 25, 50 and 75% indicate low, medium and high heterogeneity respectively [18].

If I² values were 50–100 indicating high heterogeneity the DerSimonian and Laird random effects method is suggested [19, 20]. When study heterogeneity was low with I² values 0–50 then the Mantel–Haenszel fixed effects method was used, according to guidelines [20, 21]. The DerSimonian and Laird random effects model was used for one polymorphism, all remaining studies were homogenous, therefore the Mantel–Haenszel fixed effects model was used.

Sensitivity analysis was conducted on suitable polymorphisms (>3 studies).

Publication bias was assessed using funnel plot asymmetry [22]. Egger’s test was not performed in accordance with guidelines from Sterne et al. [23, 24].

Subgroup-analyses were conducted if there were greater than 3 studies per subgroup of gender, ethnicity or MMR mutation subtype. Sub-group analyses were possible for gender for two single nucleotide polymorphisms (SNPs).

## Results

Sixty four polymorphisms in 39 genes were eligible for inclusion. Insufficient data for meta-analysis was available for 53 polymorphisms. This was due to a small number of studies being present for each SNP, with only one study available for 45 polymorphisms and two available for eight polymorphisms. Polymorphisms which could not be analysed are not presented in this paper but are available upon request.

Out of the 53 excluded polymorphisms 48 showed no significant difference, while five demonstrated a significant change in ORs. These were for *CYP17A1* (rs743572), *KIF20A* (rs10038448), *CDC25C* (rs6874130), *KDM3B*/*FAM53C* (rs3734168) and *SMAD7* (rs4939827).

For most polymorphisms, studies were homogenous. However two polymorphisms displayed heterogeneity—*GSTT1* (null variant) displayed a low level of heterogeneity (I² = 13.92) and *CYP1A1* (rs4646903) displayed a high level of heterogeneity (I² = 77.07).

It was possible to conduct meta-analyses on ten polymorphisms in five genes and five un-associated SNPs. Studies which were eligible and were included in the meta-analysis are shown in Table 2. Details on included polymorphisms are shown in Table 3.

**Table 2:** Individual study ORs, 95% CI and the calculated MAF

Gene	Polymorphism	Minor allele	MAF	OR (95% CI)	Study
*P53*	Rs1042522	C	0.22	1.16 (0.64–2.11)	Chen et al. [25]
	Rs1042522	C	0.23	0.87 (0.56–13.6)	Talseth et al. [26]
	Rs1042522	C	0.21	1.04 (0.63–1.72)	Sotamaa et al. [27]
	Rs1042522	C	0.23	0.74 (0.37–1.47)	Jones et al. [28]
*CYP1A1*	Rs4646903	C	0.14	1.17 (0.71–1.91)	Pande et al. [29]
	Rs4646903	C	0.08	2.86 (1.31–6.21)	Talseth et al. [30]
	Rs4646903	C	0.11	0.91 (0.65–1.27)	Houlle et al. [31]
*8q23.3*	Rs1689766	C	0.07	1.34 (0.92–1.96)	Win et al. [32]
	Rs1689766	C	0.08	1.06 (0.69–1.64)	Talseth-Palmer et al. [33]
	Rs1689766	C	0.07	1.25 (0.85–1.84)	Houlle et al. [31]
	Rs1689766	C	0.10	1.10 (0.71–1.71)	Wijnen et al. [34]
*11q23.1*	Rs3802842	T	0.29	1.08 (0.88–1.33)	Win et al. [32]
	Rs3802842	T	0.26	1.09 (0.84–1.42)	Talseth-Palmer et al. [33]
	Rs3802842	T	0.28	1.03 (0.82–1.30)	Houlle et al. [31]
	Rs3802842	T	0.25	1.18 (0.87–1.60)	Wijnen et al. [34]
*8q24.1*	Rs6983267	A	0.47	0.88 (0.72–1.06)	Win et al. [32]
	Rs6983267	A	0.48	1.01 (0.80–1.27)	Talseth-Palmer et al. [33]
	Rs6983267	A	0.47	0.94 (0.72–1.23)	Wijnen et al. [34]
*10p14*	Rs10795668	C	0.30	0.98 (0.78–1.23)	Win et al. [32]
	Rs10795668	C	0.33	0.95 (0.74–1.21)	Talseth-Palmer et al. [33]
	Rs10795668	C	0.33	1.06 (0.80–1.41)	Wijnen et al. [34]
*15q13.3*	Rs4779584	T	0.22	0.96 (0.76–1.20)	Win et al. [32]
	Rs4779584	T	0.23	0.93 (0.70–1.24)	Talseth-Palmer et al. [33]
	Rs4779584	T	0.20	0.84 (0.59–1.19)	Wijnen et al. [34]
*CCND1*	Rs9344	A	0.50	1.33 (0.97–1.82)	Talseth-Palmer et al. [33]
	Rs9344	A	0.44	1.05 (0.64–1.73)	Chen et al. [25]
	Rs9344	A	0.42	1.02 (0.73–1.42)	Krüger et al. [35]
	Rs9344	A	0.57	1.37 (0.84–2.24)	Bala et al. [36]
	Rs9344	A	0.45	1.06 (0.64–1.77)	Zexevic et al. [37]
	Rs9344	A	0.44	1.01 (0.55–1.86)	Kong et al. [38]
*GSTT1*	–	Null	0.23	0.80 (0.44–1.43)	Pande et al. [33]
	–	Null	0.21	1.08 (0.56–2.08)	Talseth et al. [35]
	–	Null	0.50	1.68 (0.80–3.55)	Felix et al. [39]
*GSTM1*	–	Null	0.44	0.95 (0.58–1.56)	Pande et al. [33]
	–	Null	0.55	1.00 (0.59–1.71)	Talseth et al. [35]
	–	Null	0.15	1.40 (0.50–3.93)	Felix et al. [37]
	–	Null	0.40	0.85 (0.39–1.87)	Jones et al. [40]

*MAF* minor allele frequency, *OR* odds ratio, *CI* confidence interval

**Table 3:** Details on included polymorphisms

Gene	Polymorphism	Minor allele	Number of included studies	Pooled cases/controls	OR (95% CI)	I^2^ values (%)
*P53*	Rs1042522	C	4	345/281	0.95 (0.73–1.23)	0.00
*CYP1A1*	RS4646903	C	3	567/656	1.33 (0.72–2.46)	77.07
–	Rs1689766	C	4	1127/1768	1.20 (0.98–1.47)	0.00
–	Rs3802842	T	4	1117/1749	1.08 (0.96–1.22)	0.00
–	Rs6983267	A	3	775/1321	0.93 (0.82–1.06)	0.00
–	Rs10795668	C	3	706/1247	0.99 (0.86–1.14)	0.00
–	Rs4779584	T	3	776/1333	0.92 (0.79–1.08)	0.00
*CCND1*	Rs9344	A	6	733/461	0.87 (0.73–1.03)	0.00
*GSTT1*	–	Null	3	279/325	1.06 (0.73–1.55)	13.92
*GSTM1*	–	Null	4	321/385	0.99 (0.72–1.35)	0.00

*OR* odds ratio, *CI* confidence interval

Forest plots were created to investigate the pooled ORs, 95% CI and individual study weights. We did not find any polymorphism that was statistically significant for any change in the risk of CRC development.

### Subgroup analysis

Insufficient data was reported for subgroup analysis regarding ethnicity. However adequate data was received in order to conduct gender subgroup analysis on two polymorphisms and MLH1 gene mutation subtypes.

### Gender

Gender subgroup analysis was possible for two SNPs. These were for rs16892766 and rs3802842, and results are presented in Table 4.

**Table 4:** Results of gender subgroup analysis

Polymorphism	Minor allele	Male OR (95% CI)	Female OR (95% CI)	Male vs. Female OR (95% CI)
Rs1689766	C	1.53 (1.12–2.10)	0.98 (0.68–1.39)	1.68 (1.18–2.41)
Rs3802842	T	1.15 (0.95–1.41)	1.01 (0.83–1.22)	1.16 (0.94–1.43)

*OR* odds ratio, *CI* confidence interval

The overall OR for rs16892766 is not significant (OR 1.20, 95% CI 0.98–1.47). However when subgroup analysis was conducted on the individual genders the OR for males moved into being statistically significant. When a comparison was made between males and females positive for a diagnosis of CRC it was indicated that males with this polymorphism will have a higher risk of developing CRC than females. It could be inferred from this data that the minor allele (C) increases the risk of males developing CRC and that the risk allele for this polymorphism is C. No significant association was found with the polymorphism rs3802842 for either males or females.

### MMR gene mutation subtypes

Analysis was possible for the SNPs rs16892766 and rs3802842. This was possible through extraction of data from Talseth-Palmer et al. [13] which involved a combined analysis of Talseth-Palmer et al [33] and Wijnen et al. [33, 34, 41]. Further data was extracted from Win et al. [32]. ORs failed to reach significance for both SNPs analysed in the *MLH1* with values for rs16892766 being OR 1.12, 95% CI 0.79–1.59, values for rs3802842 being OR 1.22, 95% CI 0.97–1.53.

### Sensitivity analysis

Sensitivity analysis was possible for five polymorphisms; *P53* (rs1042522), *CCND1* (rs9344), *GSTM1*, rs16892766 and rs3802842. Each study was removed in succession and ORs and 95% CI values were recalculated. No significant changes in pooled ORs, 95% CI or study heterogeneity were observed.

### Publication bias

**Fig. 2:**
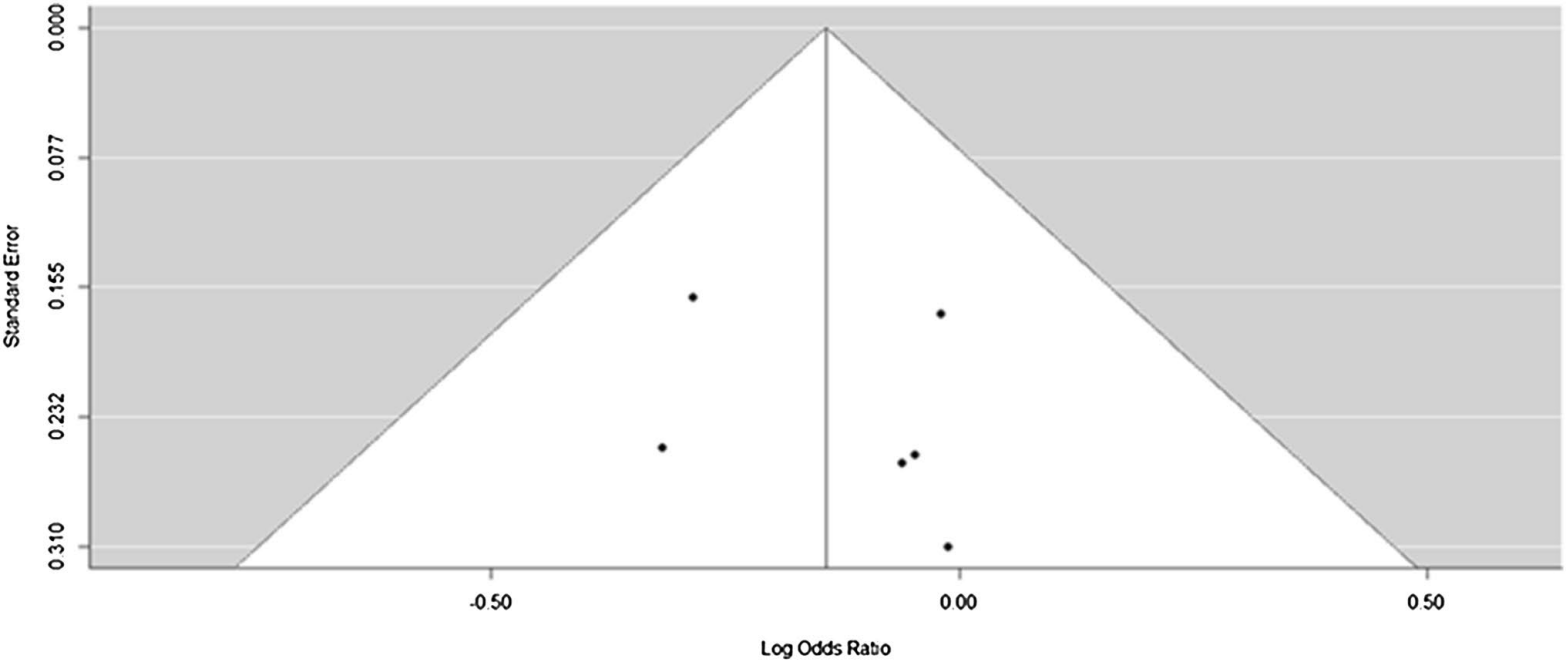
Funnel plot of polymorphism rs9344 in the gene *CCND1*

Nine of the polymorphisms studied did not show publication bias and showed symmetry in a funnel plot. The funnel plot for *CCND1* is shown in Fig. 2. However the gene *CYP1A1* was an anomaly and was asymmetrical. Due to the low number of studies included in the analysis the study bias is difficult to predict.

## Discussion

The aim of this study was to investigate the association between genetic risk modifiers and the risk of developing CRC in a LS patient cohort. The results indicate that polymorphism rs16892766 is statistically significant in males, with the minor allele (C) increasing the risk of development of CRC. The rest of the polymorphisms investigated did not achieve significance, suggesting that the role played by these risk modifiers is a minor one. However due to some polymorphisms being close to significance more study into this area and a larger data pool would help to provide increased certainty into any possible role including greater insight into specific MMR gene mutation subtypes to discern whether the minor role still holds true.

### Meta-analysis

P53 functions as a tumour suppressor gene regulating cell cycle control, apoptosis and DNA integrity [42]. Results did not show a significant association in developing CRC. Whilst there have not been any meta-analyses looking at this polymorphism in LS patients it is in broad agreement with other literature sources that have investigated this polymorphism in non LS cohorts [43]. Studies included tended to be of a Caucasian ethnic background, further studies looking at various other populations could lead to other results.

The *CCND1* gene encodes for Cyclin D1 which forms the regulatory subunit of CDK4/6 enzyme. This can cause tumourgenesis by directly phosphorylating the tumour suppressor protein retinoblastoma (Rb); this leads to cells being more likely to bypass the G1 to S phase cell cycle checkpoint which leads to cancer development [43]. There was no significant association with CRC risk in the *CCND1* polymorphism (rs9344) which is consistent with other literature sources. Zhang et al. conducted a meta-analysis on *CCND1* and CRC susceptibility [44]. They included LS patients as part of their study and were not able to find any increased CRC risk in LS patients using the dominant, co-dominant and recessive models, whereas in this study the allele specific model was used [44]. Though this study did not find an association, results were very close to significance. Further studies providing comparisons for MMR gene mutation subtypes and ethnicities would help determine if any group attained significance for CRC development.

**Fig. 3:**
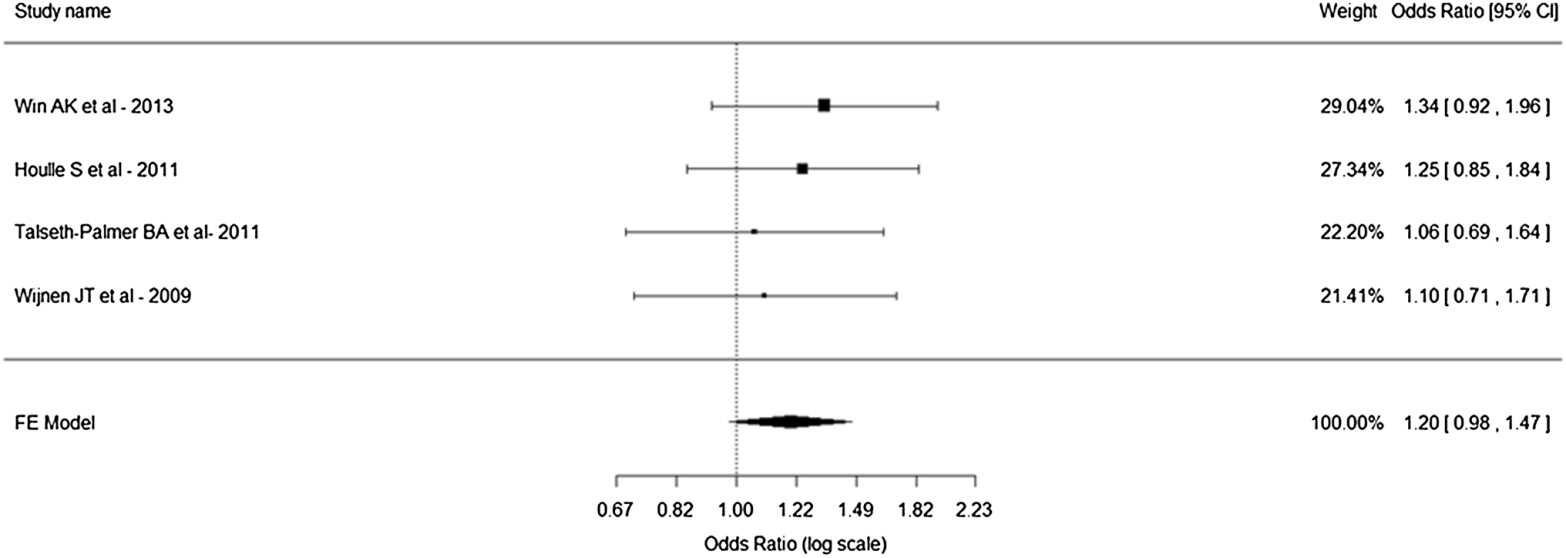
Forest plot of polymorphism rs16892766 (8q23.3)

Rs16892766 overall did not reach significance; however results were very close to reaching significance, the forest plot of this is shown in Fig. 3. Combined analyses of some, but not all of the studies included in this study indicated that there was no altered risk of CRC development in LS [45]. This was comparable to the results for rs3802842 where overall no significant results were observed. It has been observed that *MLH1* mutation carriers with this polymorphism do have an increased risk of developing CRC [41]. Overall in other combined analyses unadjusted values did not reach significance, however when adjusted for gene, gender and country of sample origin, results were significant [41]. Our analysis of the combined data sets did not discover a significant relationship in this particular SNP. It must be noted however that results were very close to reaching significance in this instance and further evidence should be obtained to achieve greater certainty in the role rs3802842 plays in *MLH1* mutation carriers. There has been no evidence of *MSH2* carriers having an altered risk due to rs16892766 or rs3802842 mutations. The other three polymorphisms (rs6983267, rs10795668, rs477958) that were obtained via GWAS studies did not alter risk of CRC development.

*GSTM1* and *GSTT1* are two subtypes of the Glutathione-S-transferase family of enzymes that can protect against developing cancer [45]. They are thought to play a role in electrophile detoxification by glutathione conjugation and by modulating other enzymes’ functions such as DNA repair and therefore preserving genomic integrity [46, 47]. In *GSTM1* the null variant is the most common polymorphism, which results in reduced enzymatic activity and has been associated with the development of cancers [45]. Previous meta-analyses have reported an association with CRC development; however this was not in LS patients. Cai et al. reported increased risk in Asians (OR 1.14, 95% CI 1.013–1.29), and Economopoulos et al. in Caucasians (OR: 1.150, 95% CI: 1.060–1.248) but were unable to find significant results in a Chinese cohort [45, 48]. This study differs from the other literature sources, however due to the very low changes in OR, and the small number of studies included in this analysis it is possible that detection of an association was not made. Also, the association may only be present in non LS patients and more research is needed as to whether this is the case.

For the gene *GSTT1* we did not find an association between risk of CRC development and the null variant. There was a small amount of study heterogeneity (I² = 13.92) which was classified as low study heterogeneity. It was not appropriate to remove any studies increasing heterogeneity due to only three studies being present. Results were similar to what was found for *GSTM1*, where some meta-analyses have found an association with very low 95% CI [48, 49]. Further insight is needed into the disparity between LS and non LS cohorts.

No association was found between *CYP1A1* and CRC development. As only three studies were included it was not possible to conduct sensitivity analysis on the data to address the high heterogeneity. Zheng et al. concluded that this polymorphism of *CYP1A1* is a low penetrance modifier of CRC development [50]. Due to the small number of studies included and the high heterogeneity between them it is difficult to draw a conclusion based on the results obtained.

Subgroup analysis for gender was possible for two of the polymorphism (rs16892766, rs3802842). The rs16892766 polymorphism when analysed for males was significant. However for females there remained no effect. When a direct comparison was made between males and females, results were significant for an increased OR for males. The polymorphism has not been mapped to any particular gene, although it has been mapped to a linkage disequilibrium block that contains the gene *EIF3H* [51]. The *EIF3H* gene codes for the H subunit of eukaryotic translation initiation factor 3 (*EIF3*). It is thought to be involved in protein synthesis and overexpression results in increased proliferation, growth and cell survival [52].

### Systematic review

Candidate polymorphisms were often not included in the meta-analysis due to a small number of studies investigating their effect in a LS patient population. They were still found in the systematic review and data was extracted from them and analysed. Four studies were found with statistically significant results and are shown in Table 5.

**Table 5:** Significant studies not included in meta-analysis

Gene	Polymorphism	Minor allele	MAF	OR (95% CI)	Study
*CYP17A1*	Rs743572	C	0.39	1.83 (1.11–3.00)	Campbell et al. [53]
*KIF20A*	Rs10038448	G	0.21	1.46 (1.07–1.99)	Chen et al. [54]
*CDC25C*	Rs6874130	C	0.22	1.47 (1.08–2.00)	Chen et al. [54]
*KDM3B/FAM53C*	Rs3734168	A	0.17	1.40 (1.00–1.96)^a^	Chen et al. [25]
*SMAD7*	Rs4939827	T	0.49	1.27 (1.04–1.57)	Win et al. [32]

*MAF* minor allele frequency, *OR* odds ratio, *CI* confidence interval

^a^Although the 95% CI for *KDM3B/FAM53C* is 1.00–1.96 the actual 95% CI did cross 1, however upon rounding to 2 d.p. the original value was rounded up

Campbell et al. investigated a *CYP17A1* polymorphism (rs743572) and found it to increase risk of development of CRC [53]. However, only LS patients with a germline mutation in *MSH2* were included in the study. *CYP17A1* belongs to the cytochrome p450 family and is involved in the metabolism of endogenous compounds and sex hormones and has been associated with cancers [55].

Chen et al. found polymorphisms in *DM3B*/*FAM53C* (rs3734168) and *CDC25C* (rs6874130) to be significantly associated with the development of CRC [54]. However, both were found to be in high linkage disequilibrium with a polymorphism in *CDC25C* (rs3734166) and the possibility of being correlated with it. Win et al. observed a statistically significant association between a polymorphism in *SMAD7* (rs4939827) and CRC development [32]. *SMAD7* has effects on the TGF-b pathway. TGF-b acts as a tumour suppressor in early states of tumour formation [56]. Another study identified during the systematic review by Talseth-Palmer et al. studied the same polymorphisms and did not find a statistically significant association [33]. The effect of *SMAD7* in CRC development for LS should be investigated further due to conflicting data.

### Limitations

A large number of candidate polymorphisms could not be included due to a lack of sufficient eligible number of studies. For the polymorphisms that did qualify for inclusion, there were still only a relatively small number of studies.

Some studies had a small number of participants and were excluded for not meeting the inclusion criteria.

Another difficulty encountered was that data was frequently missing from studies. On occasion these were the genotype frequencies, meaning that some studies did not meet the inclusion/exclusion criteria on these grounds. More commonly data was missing in the form of MMR mutation subtypes and information regarding ethnicity of study participants.

## Conclusions

Overall this study did not identify any statistically significant association in ten polymorphisms for the development of CRC in LS patients. However, upon conducting gender specific sub group analysis one polymorphism (rs16892766) at chromosome locus 8q23.3 was significant for increasing the risk of males with the minor allele C. For this polymorphism it was found that C is the risk allele. The variable phenotype presentation of the disease still remains largely unexplained, and further investigation is warranted. Other factors could also be influencing the high variability of the disease, with environmental factors, copy number variants and epigenetic changes possibly having an influence, and investigation into these areas is also needed. Another factor we were not able to fully study were gene–gene interactions whereby patients with multiple low penetrance SNPs could be experiencing an additive effect to increase risk. However we conclude that there is currently no consistent evidence that the phenotype of Lynch syndrome is influenced by the effects of low penetrance modifiers.
